# How we treat NK/T-cell lymphomas

**DOI:** 10.1186/s13045-022-01293-5

**Published:** 2022-06-03

**Authors:** Eric Tse, Wei-Li Zhao, Jie Xiong, Yok-Lam Kwong

**Affiliations:** 1 Department of Medicine, Professorial Block, Queen Mary Hospital, Pokfulam Road, Hong Kong, China; 2 Shanghai Institute of Hematology, State Key Laboratory of Medical Genomics, National Research Center for Translational Medicine at Shanghai, Ruijin Hospital, Shanghai Jiao Tong University School of Medicine, Shanghai, China

**Keywords:** NK/T-cell lymphoma, Nasal, Non-nasal, Aggressive leukaemia/lymphoma, Asparaginase, Radiotherapy, Immune checkpoint, PD1

## Abstract

Natural killer (NK)/T-cell lymphomas are aggressive malignancies with a predilection for Asian and South American populations. Epstein–Barr virus (EBV) infection in lymphoma cells is universal. Predominantly extranodal, NK/T-cell lymphomas are divided clinically into nasal (involving the nose and upper aerodigestive tract), non-nasal (involving the skin, gastrointestinal tract, testes, and other organs), and aggressive leukaemia/lymphoma (involving the marrow and multiple organs) subtypes. Initial assessment should include imaging with positron emission tomography computed tomography (PET/CT), quantification of plasma EBV DNA as a surrogate marker of lymphoma load, and bone marrow examination with in situ hybridization for EBV-encoded small RNA. Prognostication can be based on presentation parameters (age, stage, lymph node involvement, clinical subtypes, and EBV DNA), which represent patient factors and lymphoma load; and dynamic parameters during treatment (serial plasma EBV DNA and interim/end-of-treatment PET/CT), which reflect response to therapy. Therapeutic goals are to achieve undetectable plasma EBV DNA and normal PET/CT (Deauville score ≤ 3). NK/T-cell lymphomas express the multidrug resistance phenotype, rendering anthracycline-containing regimens ineffective. Stage I/II nasal cases are treated with non-anthracycline asparaginase-based regimens plus sequential/concurrent radiotherapy. Stage III/IV nasal, and non-nasal and aggressive leukaemia/lymphoma cases are treated with asparaginase-containing regimens and consolidated by allogeneic haematopoietic stem cell transplantation (HSCT) in suitable patients. Autologous HSCT does not improve outcome. In relapsed/refractory cases, novel approaches comprise immune checkpoint blockade of PD1/PD-L1, EBV-specific cytotoxic T-cells, monoclonal antibodies, and histone deacetylase inhibitors. Future strategies may include inhibition of signalling pathways and driver mutations, and immunotherapy targeting the lymphoma and its microenvironment.

## Introduction

Lymphomas arising putatively from natural killer (NK) cells were first reported more than seventy years ago [1]. Variously referred to as polymorphic reticulosis, lethal midline granuloma, and angiocentric T-cell lymphoma [2, 3], most of these lymphomas show features typical of NK-cells, being negative for surface CD3; positive for cytoplasmic CD3 epsilon (ε), CD56, and cytotoxic molecules (perforin, granzyme B, or TIA1); with T-cell receptor (*TCR*) gene in germline configuration [4, 5]. Epstein–Barr virus (EBV) infection of the lymphoma cells is universal, which can be detected by in situ hybridization (ISH) for EBV-encoded small RNA (EBER). In a minority of cases, the lymphoma is of bona fide T-cell lineage, being positive for surface CD3, CD56, cytotoxic molecules, and possesses clonally rearranged *TCR* genes [5]. The current World Health Organization (WHO) lymphoma classification adopts the nomenclature of NK/T-cell lymphoma to reflect its possible NK-cell or T-cell origin [6].

**Fig. 1:**
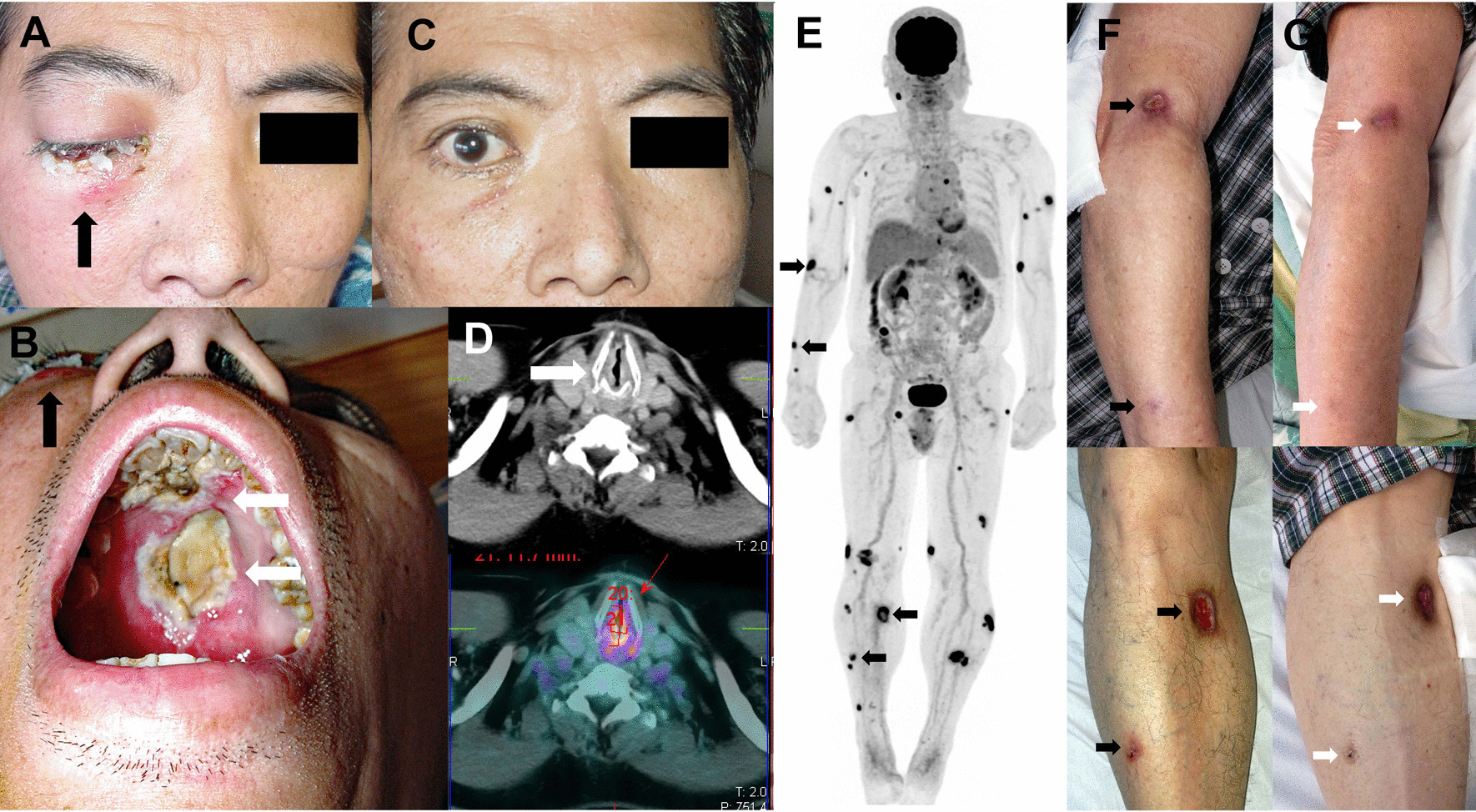
Clinical subtypes of NK/T-cell lymphomas. **a** Nasal NK/T-cell lymphoma with superior invasion into the right orbit, leading to extensive swelling and scabbing (arrow). **b** Same case with right orbital invasion (black arrow). Inferior invasion resulted in extensive necrosis and almost complete destruction of the hard palate (white arrows). The ensuing perforation of the hard palate would lead to a communication between the nasal and oral cavities, giving rise to the classical “lethal midline granuloma”. **c** Same patient about two weeks after commencement of the first cycle of the SMILE regimen. There was rapid and complete resolution of the right orbital swelling and scabbing. **d** Another case of upper aerodigestive tract NK/T-cell lymphoma. There was extensive involvement of the subglottis (arrow), which was markedly hypermetabolic on positron emission tomography computed tomography (PET/CT). Note that the larynx was reduced to a mere slit, causing nearly fatal airway obstruction that necessitated emergency tracheostomy. **e** A case of apparent non-nasal NK/T-cell lymphoma with extensive skin involvement, which on PET/CT scan was shown as numerous hypermetabolic cutaneous deposits (arrows). Examination of the nasopharynx did not show any obvious lesion. However, blind biopsies showed nasopharyngeal involvement, rendering this case indistinguishable from nasal NK/T-cell lymphoma with extensive cutaneous metastases. **f** Cutaneous lesions of the same case, with arrows indicating deposits corresponding to those shown by arrows on the PET/CT (**e**). **g** After the first cycle of an asparaginase-containing regimen, showing complete healing of the skin lesions (arrows)

NK/T-cell lymphomas show a strong predilection for Asian and South American populations [2, 4, 7], although cases from Western populations are increasingly reported [8]. Clinically, NK/T-cell lymphomas are predominantly extranodal. In about 80% of cases, the initially involved sites are the nasal cavity, paranasal sinuses, nasopharynx, oropharynx, and upper aerodigestive tract. Collectively, they are referred to clinically as the nasal subtype (Fig. 1a–d). In about 15–20% of cases, the primary presentation sites include the skin, gastrointestinal tract, testicles, and salivary glands. Collectively, they are referred to clinically as the non-nasal subtype (Fig. 1e–g). Notably, these sites are also where nasal NK/T-cell lymphomas metastasize to. In < 5% of cases, the lymphoma may be disseminated on presentation with hepatosplenomegaly, lymphadenopathy, marrow involvement, and a leukaemia phase. These disseminate cases are referred to clinically as the aggressive lymphoma/leukaemia subtype [2, 5] and pathologically as aggressive NK-cell leukaemia by the WHO classification [9].

## Diagnostic evaluation and differential diagnoses

WHO diagnostic criteria stipulate that, in addition to standard histopathologic features, NK/T-cell lymphoma must be EBV+, and express either CD56 or cytotoxic molecules. If both CD56 and cytotoxic molecules are negative, the diagnosis becomes EBV+ peripheral T-cell lymphoma [6]. Four clinicopathologic entities should be distinguished from NK/T-cell lymphomas. Plasmacytoid dendritic neoplasms, previously erroneously referred to as blastoid NK-cell lymphomas, are cutaneous CD56+ neoplasms. They are, however, negative for CD3, cytotoxic molecules, and EBV [7]. NK-cell lymphomatoid gastropathy/NK-cell enteropathy is a rare, apparently non-neoplastic proliferation of NK-cells in the stomach, and small and large bowels [10, 11]. They are EBV-negative and self-limiting. Chronic lymphoproliferative disorder of NK-cells is uncommon and of uncertain reactive or neoplastic nature. They are EBV-negative. Exceptional cases of EBV-negative aggressive leukaemia/lymphoma of putative NK-cell derivation have been reported [12]. With very few cases described, it is uncertain whether they are related to NK/T-cell lymphomas.

## Molecular alterations and implications on treatment

Molecular alterations in NK/T-cell lymphoma may have potential implications on therapeutic approaches (Table 1). Chromosome 6q21–25 deletion is a recurrent aberration, resulting in loss of putative tumour suppressor genes [7, 13, 14]. Gene expression profiling and genomic investigations also identified losses and gains of chromosomal regions, resulting in putative dysregulations of genes related to multiple cellular processes, signal transduction, and immune functions (Table 1) [15, 16]. Moreover, aberrations of non-coding RNAs leading to gene deregulation were observed, which might result in epigenetic alterations [17]; downregulation of tumour suppressors [18], and aberrant p-STAT3 expression [19]. Next-generation sequencing further defined recurrent mutations in RNA helicases, tumour suppressors, JAK-STAT pathway genes, epigenetic modifiers, and other oncogenes [16, 20, 21]. These alterations might provide clues for the search of driver events or mutations amendable to therapeutic intervention.

**Table 1:** Molecular alterations and their potential implications on therapeutic targeting

Functional pathways	Genes involved (references)	Potential targeting*
Tumour suppression	*PRDM1*, *FOXO3*, *HACE1* (7), *CAV1*, *CAV2*, *DLC1* (15)	Specific gene targeting
Oncogenesis	*NOTCH3*, *KRAS*, *BRAF*, *PRKD1*, *MAP3K5*, *PTPRK* (16, 20, 21); *DDX3X* (16, 21)	Specific gene targeting
Multi-function pathway activation	*PRKCQ*, *TNFRSF21*, *CUL1*, *FSD1*, *SGK1* (15, 16)	NF-κB, WNT signalling
	*AKT3*, *IL6R*, *CCL2* (15, 16); *JAK3, STAT3, STAT5B* (16, 20, 21)	JAK/STAT signalling
Derangement of tissue proliferation	*CCND3* (15); *S100A16*, *LAMB1*, *LAMC1*, *COL1A2*, *CTSB* (15, 16)	Cell cycle
	*MET, S100A13* (15)	Angiogenesis
Epigenetic dysregulation	*TP73*, *RARB*, *P15*, *P16*, *PRDM1*, *ATG5*, *AIM1*, *BCL2L11*, *DAPK1*, *TET2*, *PTPN6*, *SOCS6*, *PTPRK*, *ASNS* (25, 26); *KMT2D*, *KMT2C*, *BCOR*, *EP300*, *HDAC9*, *ARID1A*, *ASXL3* (16, 20,21); *EZH2* (17)	Epigenetic modifiers
Immune escape	*PD-L1*, *PD-L2* (15, 16)	Immune checkpoints

*Potential strategies that may or may not yet be supported by experimental or clinical data

Genome-wide association studies in predominantly Asian patients identified three susceptibility loci, *HLA-DPB1*, *IL18RAP*, and *HLA-DRB1*, with genetic differences leading to amino-acid changes potentially affecting immune responses to EBV infection, thus contributing to the pathogenesis of and increasing the susceptibility to NK/T-cell lymphoma [22, 23]. These findings suggest that targeting EBV might be a treatment strategy [24].

Epigenetic derangements are another key feature in NK/T-cell lymphomas. Hypermethylation of promoter regions of genes involved in tumour suppression, apoptosis, inflammation, and metabolism was consistently shown [25, 26]. Therefore, epigenetic modifiers might be therapeutically useful [27].

A multi-omics approach has shown therapeutic leads [28]. Transcriptomics-based approaches could be used to divide NK/T-cell lymphomas into three subtypes, viz., TSIM, MB, and HEA [16]. The TSIM subtype (about 55% of cases) was defined by mutations of *TP53* and genes in the JAK-STAT pathway, amplifications of the 9p24.1/*JAK2*, 17q21.2/*STAT3*/*5B*/*5A* and 9p24.1/*PD-L1/2* loci, and 6q21 deletion. Lymphoma cells had a predominant NK-cell gene expression pattern, with JAK/STAT pathway activation, programmed cell death protein ligand 1/2 (PD-L1/2) overexpression, and genomic instability. The MB subtype (about 18% of cases) was defined by *MGA* gene mutation and loss of heterozygosity (LOH) of the 1p22.1/*BRDT* locus. Lymphoma cells were intermediate between NK-cells and T-cells in gene expression. Mutations in *MGA* led to MYC overexpression and combined with *BRDT* LOH resulted in activation of the MAPK, NOTCH, and WNT pathways. The HEA subtype (about 27% of cases) was defined by mutations in *HDAC9*, *EP300*, and *ARID1A*. Lymphoma cells had a predominant T-cell gene expression profile, overexpression of the histone chaperone DAXX, and activation of the NF-κB and TCR signalling pathways. With RNA-seq and immunohistochemical studies, the TSIM, MB, and HEA subtypes were typified by overexpression of PD-L1, MYC, and DAXX, respectively. The potential therapeutic implications might be immune checkpoint inhibitors for TSIM cases, MYC inhibitors for MB cases, and epigenetic modifiers for HEA cases.

A recent combined nanostring and immunohistochemical analysis, using FoxP3, PD-L1, and CD68 expression, divided patients into four immune microenvironment subtypes, viz., immune tolerance, immune evasion-A, immune evasion-B, and immune silenced [29]. Preliminary results showed that responses to blockade of the immune checkpoint protein programmed cell death protein 1 (PD1) might be related to these immune subtypes (1/1 for the immune tolerance group, 3/5 in the immune evasion groups, and 0/5 for the immune-silenced group).

## Pathogenetic mechanisms and implications on treatment

In NK/T-cell lymphomas, dysregulation of glutamine metabolism constitutes a metabolic vulnerability [30]. Metabolomic profiling showed that these lymphoma cells often displayed a profile of low asparagine synthetase activity, reflected by increase in serum levels of alanine, aspartic acid, glutamine, and succinic acid [30]. A low level of asparagine synthetase rendered lymphoma cells susceptible to asparaginase treatment and might be a biologic marker of treatment response and prognosis [31]. Integrative analysis of targeted serum metabolomic analysis and paired tumour RNA-seq data identified excitatory amino acid EAAT3 (encoded by *SLC1A1*) as an extracellular glutamine transporter, which increased cellular glutamine uptake and enhanced glutathione metabolic flux, thereby inducing glutamine addiction. Furthermore, SLC1A1 overexpression also downregulated PD-L1. Targeting SLC1A1-mediated glutamine addiction with asparaginase would therefore be therapeutically relevant [32].

EBV exists in a latency II state in NK/T-cell lymphoma [4, 5], with expression of the viral oncoprotein LMP1. Various mechanisms including *STAT3*/*STAT5* mutations lead to activation of the JAK/STAT pathway [5, 7]. Both LMP1 and JAK/STAT activation act on the enhancer and promoter of the *PD-L1* gene, leading to its overexpression. By interacting with the immune checkpoint inhibitor PD1 on cytotoxic cells, PD-L1 expression on lymphoma cells impairs immunosurveillance, constituting an immune escape mechanism. This process appeared further enhanced by structural rearrangements disrupting the 3’-UTR of *PD-L1* [33]. Inhibition of the PD1/PD-L1 axis is thus an attractive treatment direction.

## Assessment of newly diagnosed patients

Bone marrow aspirate may show haemophagocytosis, which on its own is not indicative of marrow involvement. Cytologically, lymphoma cells possess abundant cytoplasm with azurophilic granules. On trephine biopsy, EBER ISH is the most reliable way of defining lymphomatous infiltration [7].

NK/T-cell lymphoma is 18-fluorodeoxyglucose-avid [34, 35], so that positron emission tomography computed tomography (PET/CT) should be considered the most accurate and standard modality for radiologic staging. A clinically non-nasal case shown on PET/CT to have occult nasal involvement should be re-classified as a disseminated nasal one. Accordingly, studies of “non-nasal” NK/T-cell lymphomas not employing PET/CT as the imaging modality should no longer be considered reliable.

As NK/T lymphoma cells undergo apoptosis, EBV DNA fragments are released into the circulation [36]. Quantification of circulating EBV DNA provides a molecular measure of tumour load [37]. Whole blood is unsuitable and should not be used, because of unpredictable errors introduced by circulating memory B-cells that may be EBV-infected [36, 38]. Plasma EBV DNA is instead accurate and should be employed as a surrogate marker of lymphoma load. Presentation plasma EBV DNA measures lymphoma load and is of prognostic significance [37]. During treatment, plasma EBV DNA reflects lymphoma response and may also be of prognostic significance [39, 40].

## Prognostication of NK/T-cell lymphomas

The International Prognostic Index (IPI) remained useful for NK/T-cell lymphomas treated with conventional anthracycline-containing regimens [41]. A similar prognostic model, the Korean-IPI, was also developed for patients treated with anthracycline-containing regimens [42]. These prognostic models, although apparently still retaining significance for non-anthracycline-containing regimens, have since been superseded. Two prognostic models, CA staging [43] and NRI scoring [44], based partly on negative scoring for lymphoma local invasion and the non-nasal subtype, have been proposed. The problems of these models are the unclear and subjective definition of lymphoma local invasion, and the lack of the use of PET/CT in patient evaluation, thereby making classification of non-nasal cases insecure. More recently, a score based on polymorphism of seven single nucleotides was described to be prognostic [45]. A clear biologic basis of how single nucleotide polymorphisms impact on lymphoma response and prognosis is not apparent, and the test is not readily available, so that the model is of limited value in routine practice.

A more practical prognostic model developed for patients treated with non-anthracycline-containing regimens is the prognostic index for NK/T-cell lymphomas (PINK) (negative scoring parameters: age > 60 years, stage III/IV disease, distant lymph node involvement, non-nasal subtype), and its variant PINK-E (additional negative scoring parameter: detectable presentation EBV DNA) [46]. PINK-E appears particularly useful, as it incorporates a biologic parameter, EBV DNA, which has been shown to be prognostic important [36–40].

Prognostic models based on presentation parameters rely predominantly on initial lymphoma load and location. Response to treatment is not assessed, so that such models cannot inform treatment dynamically. Instead, two parameters that evolve during treatment, circulating plasma EBV DNA, and PET/CT, may be more useful. Interim plasma EBV DNA [39] and PET/CT [47] after two to three cycles of treatment had been shown to predict the ultimate outcome. At interim, patients with undetectable plasma EBV DNA and PET/CT of Deauville score ≤ 3 had outcome significantly superior to those with detectable EBV DNA or PET/CT of Deauville score> 3 [39, 47]. Similarly, end-of-treatment detectable circulating EBV DNA and PET/CT scan with a Deauville score of > 3 had also been shown to portend inferior long-term prognosis [40], suggesting that patients with such results would require additional treatment to improve outcome.

In practice, PINK / PINK-E is the preferred prognostic model at initial diagnosis. Based on risk stratification, protocol/trial-driven triage of patients to different asparaginase-containing regimens can be adopted. During treatment and particularly at interim after two to three cycles of therapy, assessment of plasma EBV DNA and PET/CT offers a dynamic means of prognostication. For satisfactory interim results, there are currently no data to support abbreviation of pre-planned treatment, so a conventional six-cycle strategy should be continued. However, for unsatisfactory interim results, protocol/trial-driven alteration or intensification of treatment is pertinent. Finally, at the end-of-treatment, if undetectable plasma EBV DNA and PET/CT scan with Deauville score of ≤ 3 cannot be achieved, the prognosis is poor and salvage treatment should be considered.

## Principles of treatment

NK-cells express high levels of P-glycoprotein, leading to a multidrug resistance (MDR) phenotype [48]. Anthracycline-containing (CHOP, cyclophosphamide, adriamycin, vincristine, prednisolone; or CHOP-like) regimens, designed for conventional high-grade B-cell lymphomas, are MDR-dependent and ineffective [2, 7]. Hence, various non-anthracycline-containing regimens have been developed for NK/T-cell lymphomas [2]. A central component of these regimens is asparaginase, which induces apoptosis of NK-cells in vitro [49]. Asparaginase also showed single-agent activity in relapsed/refractory NK/T-cell lymphoma [2, 7]. Practically every effective regimen currently used in NK/T-cell lymphoma contains asparaginase or its pegylated form (Table 2).

**Table 2:** Major regimens used in NK/T-cell lymphomas, listed in alphabetical order according to acronyms

Regimens	Drugs and schedule	References
AspaMetDex	*E. coli* L-asparaginase: 6000 U/m^2^, IM, days 2, 4, 6, 8	[73]
	Methotrexate: 3000 mg/m^2^, IV, day 1	
	Dexamethasone: 40 mg, oral, days 1–4	
DDGP	Dexamethasone: 15 mg/m^2^, IV, days 1–5	[76]
	Cisplatin: 20 mg/m^2^, IV, days 1–4	
	Gemcitabine: 800 mg/m^2^, IV, days 1, 8	
	Pegaspargase: 2500 IU/m^2^, IM, day 1	
DeVIC (2/3)	Dexamethasone: 40 mg, IV, days 1–3	[57]
	Etoposide: 67 mg/m^2^, IV, days 1–3	
	Ifosfamide: 1000 mg/m^2^, IV, days 1–3	
	Carboplatin: 200 mg/m^2^, IV, day 1	
DICE-L-asp	Dexamethasone: 20 mg/m^2^, days 1–4	[69]
	Ifosfamide: 1200 mg/m^2^, IV, days 1–3	
	Cisplatin: 25 mg/m^2^, IV, days 1–4	
	Etoposide: 60 mg/m^2^, days 1–4	
	L-asparaginase: 6000 U/m^2^, days 6–11	
GELAD	Gemcitabine: 1000 mg/m^2^, IV, day 1	[70]
	Etoposide: 60 mg/m^2^, IV, days 1–3	
	Pegaspargase: 2000 U/m^2^, day 5	
	Dexamethasone: 40 mg, days 1–4	
GELOX	Gemcitabine: 1000 mg/m^2^, IV, days 1, 8	[66]
	*E. coli* L-asparaginase: 6000 U/m^2^, IM, days 1–7	
	Oxaliplatin: 130 mg/m^2^, IV, day 1	
LVP	L-asparaginase: 6000 IU/m^2^, IV, days 1–5	[63]
	Vincristine: 1.4 mg/m^2^, IV, day 1	
	Prednisolone: 100 mg, oral, days 1–5	
MEDA	Methotrexate: 3000 mg/m^2^, IV, day 1	[74]
	Etoposide: 100 mg/m^2^, IV, days 2–4	
	Dexamethasone: 40 mg, IV, days 2–4	
	Pegaspargase: 2500 U/m^2^, day 4	
MESA	Methotrexate: 1000 mg/m^2^, IV, day 1	[30]
	Etoposide: 100 mg/m^2^, days 2–4	
	Dexamethasone: 40 mg, IV, days 2–4	
	Pegaspargase: 2500 U/m^2^, IM, day 4	
P-GEMOX	Pegaspargase: 2500 IU/m^2^, IM, day 1	[67]
	Gemcitabine: 1000 mg/m^2^, IV, days 1, 8	
	Oxaliplatin: 130 mg/m^2^, IV, day 1	
SMILE	Dexamethasone: 40 mg, IV or oral, days 2–4	[62]
	Methotrexate: 2000 mg/m^2^, IV, day 1	
	Ifosfamide: 1500 mg/m^2^, IV, days 2–4	
	*E. coli* L-asparaginase: 6000 U/m^2^, IV, days 8, 10, 12, 14, 16, 18, 20	
	Etoposide: 100 mg/m^2^, IV, days 2–4	
VIDL	Etoposide: 100 mg/m^2^, IV, days 1–3	[60]
	Ifosfamide: 1200 mg/m^2^, IV, days 1–3	
	Dexamethasone: 40 mg, IV, days 1–3	
	L-asparaginase: 4000 IU/m^2^, IM, days 8, 10, 12, 14, 16, 18, 20	
VIPD	Etoposide: 100 mg/m^2^, IV, days 1–3	[59]
	Ifosfamide: 1200 mg/m^2^, IV, days 1–3	
	Cisplatin: 33 mg/m^2^, IV, days 1–3	
	Dexamethasone: 40 mg, IV or oral, days 1–4	

IV: intravenous, IM: intramuscular; doses given are daily dosages

## Management of stage I/II nasal NK/T-cell lymphomas

**Fig. 2:**
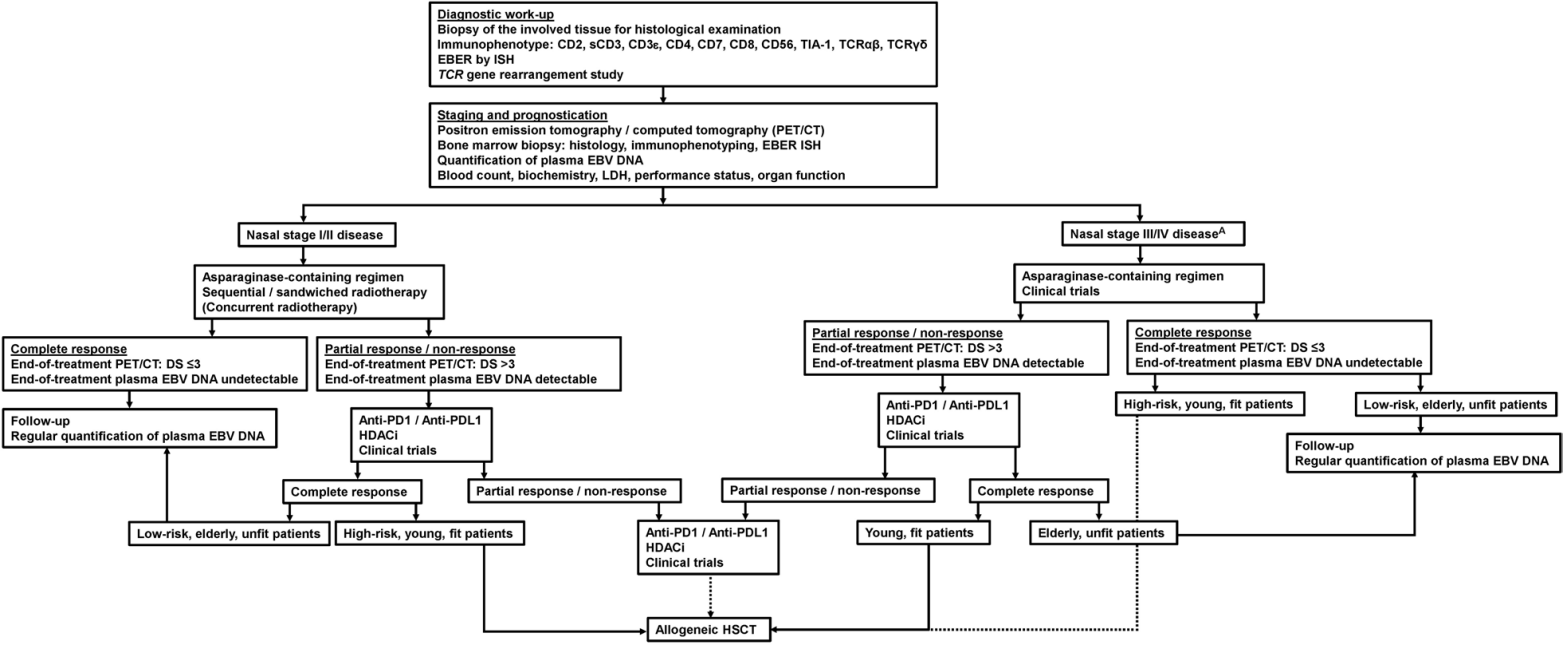
Treatment algorithm of NK/T-cell lymphoma. A denotes that for non-nasal cases of all stages, and aggressive leukaemia/lymphoma, treatment should be the same as stage III/IV nasal lymphomas. Dotted lines indicate possible options. For abbreviations please refer to the main text

Involved field radiotherapy and chemotherapy are the currently recommended treatment modalities for stage I/II nasal NK/T-cell lymphomas (Fig. 2) [50]. No prospective randomized trials have been conducted to compare how these two modalities should be sequenced or combined. Hence, centres often adopt protocols according to their expertise or the availability of timely radiotherapy.

## Radiotherapy

NK/T-cell lymphomas are radiosensitive. In stage I/II disease, the use of radiotherapy had led to better results and survival [51, 52]. Adequate doses of radiotherapy coupled with modern delivery techniques further improved outcomes. Radiotherapy doses of below 50 Gy resulted in more locoregional relapses [8, 44, 53]. The use of intensity-modulated radiotherapy (IMRT) decreased the radiation exposure to normal surrounding tissue and provided good tumour target coverage [54]. In a retrospective analysis of stage I/II nasal diseases, IMRT with or without chemotherapy, compared with 3-dimensional conformal radiotherapy, resulted in significantly better 5-year progression-free survivals (PFS; 68.9% vs. 58.2%) and overall survivals (OS; 75.9% vs. 67.6%) [55]. However, radiotherapy alone for stage I/II NK/T-cell lymphoma is associated with high systemic relapse rates. Hence, radiotherapy as a single modality should not be adopted. The only situation where radiotherapy might be used alone is in elderly patients with poor performance and significant comorbidities that preclude chemotherapy [50].

## Concurrent chemoradiotherapy

Concurrent chemoradiotherapy has been proposed for stage I/II diseases, predicated on the notion that radiosensitivity can be enhanced with concurrent chemotherapy [56]. Three regimens, DeVIC (dexamethasone, etoposide, ifosfamide, and carboplatin), VIPD (etoposide, ifosfamide, cisplatin, and dexamethasone), and the VIDL (etoposide, ifosfamide, dexamethasone, and L-asparaginase) had been used concurrently with radiotherapy in stage I/II diseases (Tables 2 and 3). For DeVIC + 50 Gy radiotherapy, the overall response rates (ORRs) were 78–89% with complete remission (CR) rates of 75–82% [57, 58]. The 5-year PFS and OS were 61–67% and 72–73%, respectively [57, 58]. For VIPD + 40 Gy radiotherapy, the ORR and CR were 83.3% and 80% and the 3-year PFS and OS were 65% and 86% [59]. The results of VIDL + 40 Gy radiotherapy were comparable, with ORR and CR of 90% and 87%, and 5-year PFS and OS of 60% and 73% [60] (Table 3).

**Table 3:** Outcome of patients with NK/T-cell lymphomas treated with asparaginase-containing regimens

Regimens	Status	Stage	ORR	CR (%)	PFS	OS	References
VIDL + RT	Newly diagnosed	I/II	90%	87	5 year: 60%	5 year: 73%	[61]
LVP + RT	Newly diagnosed	I/II	89%	81	5 year: 64%	5 year: 64%	[64]
GELOX + RT	Newly diagnosed	I/II	96%	74	5 year: 74%	5 year: 85%	[67]
P-GEMOX [+ RT for stage I/II]	Newly diagnosed	I/II	94%	80	2 year: 77%	2 year: 83%	[68]
	Newly diagnosed	I/II	94%	64	3 year: 66%	3 year: 81%	[69]
	Relapsed/refractory		81%	52	3 year: 24%	3 year: 58%	[76]
DICE-L-asp	Newly diagnosed	I/II	100%	91	5 year: 82%	5 year: 89%	[70]
MESA	New diagnosed	I/II	92%	89	2 year: 89%	2 year: 92%	[30]
SMILE [+ RT for stage I/II]	Newly diagnosed	I/II	90%	69	Not reported		[73]
		III/IV	Not reported	54	4 year: 60%	5 year: 47%	
	Relapsed/refractory		77%	66	4 year: 68%	5 year: 52%	
DDGP	Newly diagnosed	III/IV	95%	71	1 year: 86%	1 year: 90%	[77]
AspaMetDex	Relapsed/refractory		78%	61	2 year: 40%	2 year: 40%	[74]
MEDA	Relapsed/refractory		77%	61	1 year: 62%	1 year: 69%	[75]
GELAD	Newly diagnosed	I/II	94%	92	2 year: 90%	2 year: 94%	[71]

ORR: Overall response rate; CR: complete remission; PFS: progression-free survival; OS: overall survival; RT: radiotherapy; VIDL: etoposide, ifosfamide, dexamethasone, L-asparaginase; LVP: L-asparaginase, vincristine, prednisolone; GELOX: gemcitabine, L-asparaginase, oxaliplatin; P-GEMOX: pegaspargase, gemcitabine, oxaliplatin; DICE-L-asp: dexamethasone, ifosfamide, cisplatin, etoposide, L-asparaginase; MESA: methotrexate, etoposide, dexamethasone, pegaspargase; SMILE: dexamethasone, methotrexate, ifosfamide, L-asparaginase, etoposide; DDGP: dexamethasone, gemcitabine, cisplatin, pegaspargase; AspaMetDex: L-asparaginase, methotrexate, dexamethasone; MEDA: methotrexate, etoposide, dexamethasone and pegylated asparaginase; GELAD: gemcitabine, etoposide, pegasparaginase, dexamethasone

## Sequential chemotherapy and radiotherapy

Sequential chemotherapy and radiotherapy involve the initial use of chemotherapy, followed by either interim or end-of-treatment radiotherapy. In this approach, it is critical to administer effective chemotherapy. Use of the ineffective anthracycline-containing regimen CHOP followed by radiotherapy led to poor outcome, with 5-year PFS and OS of merely 54% and 65% [61]. In contrast, the use of asparaginase-containing regimens (Table 3) followed by radiotherapy led to much superior results. Excellent ORRs (90–100%) and CRs (74–91%) were observed for asparaginase-containing regimens including SMILE (dexamethasone, methotrexate, ifosfamide, L-asparaginase, and etoposide) [62], LVP (L-asparaginase, vincristine, prednisolone) [63, 64], GELOX (gemcitabine, L-asparaginase, and oxaliplatin) [65, 66], P-GEMOX (pegaspargase, gemcitabine, and oxaliplatin) [67, 68], DICE-L-asp (dexamethasone, ifosfamide, cisplatin, etoposide, L-asparaginase) [69], MESA (methotrexate, etoposide, dexamethasone, and pegaspargase) [30], and GELAD (gemcitabine, etoposide, peg-asparaginase, dexamethasone) (Table 2) [70], which were followed by interim or end-of-treatment radiotherapy. Survivals were very good, with 5-year PFS ranging from 64 to 83% (Table 2). In a retrospective analysis of 303 patients with stage I/II nasal NK/T-cell lymphoma, sequential chemotherapy and radiotherapy gave CR, PFS, and OS that were comparable with those of concurrent chemoradiotherapy with or without subsequent consolidation chemotherapy [71]. Hence, provided that effective chemotherapy is used, the timing of radiotherapy does not seem critical.

## Practical approach to stage I/II nasal NK/T-cell lymphomas

The standard-of-care is asparaginase-containing regimens combined with radiotherapy. Sequential chemotherapy and radiotherapy are adopted in most centres, as shown by the abundance of studies employing this strategy. Arranging chemotherapy is logistically easier for newly-diagnosed patients who may need immediate treatment. Furthermore, with control of lymphoma after initial chemotherapy, patients often have better performance when radiotherapy is subsequently given, thus tolerating it better. Concurrent chemoradiotherapy is hardly used, owing to logistic complexity of arranging timely radiotherapy for newly diagnosed patients, and its serious mucosal and systemic toxicity when chemotherapy is also given, making it poorly tolerated, especially in elderly patients.

During treatment, plasma EBV DNA should be serially monitored. The goal is to have undetectable plasma EBV DNA after two to three cycles of chemotherapy [39]. An interim PET/CT should also be performed, and a Deauville score of ≤ 3 should be achieved [47]. Failure to achieve these interim goals suggests that alteration or modification of treatment might be needed. On completion of treatment, the goals are undetectable plasma EBV DNA and PET/CT with Deauville score of ≤ 3; both requisites for durable remission [37, 39, 40]. Failure to achieve these end-of-treatment goals indicates that additional treatment is needed to improve outcome. Plasma EBV DNA should be monitored during follow-up. With undetectable EBV DNA, surveillance PET/CT is not necessary.

## Management of stage III/IV nasal NK/T-cell lymphomas

Asparaginase-containing chemotherapeutic regimens are the standard-of-care in these patients (Fig. 2) [2, 4]. Anthracycline-containing regimens (CHOP or CHOP-like) should not be used. The regimen SMILE [62, 72] is most popular with the best cumulative experience. In newly-diagnosed stage III/IV patients treated with SMILE, CR was achieved in 40–54% of cases, with a 5-year OS of 47% [72]. Other asparaginase-containing regimens, including AspaMetDex (L-asparaginase, methotrexate, and dexamethasone) [73], MEDA (methotrexate, etoposide, dexamethasone, and pegylated asparaginase) [30, 74], and P-GEMOX [75] had also been used in newly-diagnosed stage III/IV patients (Table 2). These regimens gave variable but largely comparable results. However, most of these studies reported only short-term data, with long-term outcome unclear. None of these regimens have been compared, so that their relative efficacies are undefined. The regimen DDGP (dexamethasone, gemcitabine, cisplatin, and pegylated asparaginase) had been compared prospectively and retrospectively with SMILE. Results purportedly showed that DDGP led to better CR and survivals [76, 77]. However, these studies were seriously flawed, because the outcomes of the SMILE cohorts were exceptionally poor, which accounted for the apparent but probably erroneous superiority of the DDGP regimen. Hence, SMILE remains the current standard for stage III/IV NK/T-cell lymphomas [72, 78]. The high efficacy of asparaginase-containing regimens notwithstanding, the survival curves of these patients plateau at about 40%, suggests that additional treatment is needed to improve outcome.

## Haematopoietic stem cell transplantation (HSCT)

Frontline autologous HSCT is generally not recommended for nasal NK/T-cell lymphoma, because of its doubtful additional benefit on survivals. In a retrospective analysis of frontline autologous HSCT in NK/T-cell lymphomas, there was an improvement in CR rate after HSCT to 90% for stage I/II patients and 65.5% for stage III/IV patients. The 3-year PFS and OS were 65% and 68% for stage I/II patients and 40% and 52% for stage III/IV patients [79]. In another phase II study of stage III/IV patients, treatment with VIDL was followed by autologous HSCT. For patients who proceeded to autologous HSCT, only 47% of cases remained in remission after a short median follow-up of 31 months [80]. Although there was no direct comparison, these results did not appear to be different from those obtained with asparaginase-containing regimens alone, suggesting that autologous HSCT in these settings did not improve outcome.

Allogeneic HSCT offers a potential cure, based on a putative graft-versus-lymphoma effect. However, no randomized trial has been conducted to examine the role of allogeneic HSCT in NK/T-cell lymphomas. An early retrospective analysis of allogeneic HSCT in NK/T-cell lymphomas, treated with heterogeneous prior regimens and allografted with variable HSC sources, showed 2-year survivals of merely 30–40% [81]. However, later studies also in highly selected patients with advanced-stage or relapsed/refractory diseases showed a 5-year OS of more than 50% [82, 83]. The high treatment-related mortality shown in these studies remains a barrier for allogeneic HSCT to be recommended for all patients with advanced-stage and relapsed/refractory diseases.

## Management of non-nasal NK/T-cell lymphomas and aggressive NK-cell leukaemia/lymphoma

Most non-nasal cases previously reported had not been staged with PET/CT. Hence, it remains uncertain whether these non-nasal cases might actually be disseminated nasal cases, which could account for their apparent inferior prognosis. The two most common primary sites are the skin and gastrointestinal tract. Cutaneous NK/T-cell lymphomas are rarely localized on presentation, usually with regional nodal or distant organ involvement [84]. Prognosis appeared poor, with a 5-year OS reported to be merely 25% [84]. Gastrointestinal NK/T-cell lymphomas are mostly advanced with B-symptoms on presentation [85]. Treatment is often delayed because of surgical complications including bowel obstruction and perforation. The median OS was dismal at < 8 months [85]. Unless patients with non-nasal lymphomas truly have localized disease on PET/CT, which is highly uncommon, they ought to receive the same treatment as for stage III/IV nasal lymphoma.

Aggressive NK/T-cell leukaemia/lymphoma is extremely aggressive, with survival measured merely in weeks before the advent of effective treatment [5, 7]. These patients should be given vigorous supportive treatment and started on asparaginase-containing regimens as soon as feasible. Allogeneic HSCT is needed for any hope of survival.

## Practical approach to stage III/IV-nasal and non-nasal NK/T-cell lymphomas, and aggressive NK-cell leukaemia/lymphoma

The standard-of-care is asparaginase-containing regimens (Fig. 2). Therapeutic goals remain undetectable plasma EBV DNA and PET/CT of Deauville score ≤ 3 at interim and end-of-treatment. Central nervous system (CNS) involvement is exceptionally rare in stage I/II nasal NK/T-cell lymphomas, but may occasionally be seen in stage III/IV-nasal and non-nasal NK/T-cell lymphomas, and aggressive NK/-T cell leukaemia/lymphomas [86]. Regimens containing intermediate-dose methotrexate (SMILE or SMILE-like) significantly decreased the risk of CNS involvement [86]. Hence, patients with high PINK/PINK-E scores or disseminated non-nasal lymphomas, which are risk factors of CNS involvement [86], should receive SMILE or SMILE-like regimens.

Because of unsatisfactory survivals, patients with stage III/IV-nasal disease and non-nasal diseases of any stage and aggressive NK-cell leukaemia/lymphomas should be evaluated for additional treatment even if molecular remission (undetectable EBV DNA) or radiologic remission (PET/CT of Deauville score ≤ 3) is achieved. Autologous HSCT does not offer any additional benefit. Allogeneic HSCT should be considered, although results remain anecdotal and data on HSC source and the optimal conditioning regimens are scarce. This is clearly an area where more research and prospective studies are required.

## Management of relapsed/refractory NK/T-cell lymphomas

Patients relapsing from or refractory to anthracycline-containing regimens can still be effectively salvaged by asparaginase-containing regimens [5, 7]. However, the outcome of relapsed/refractory patients in the era of non-anthracycline containing regimens is dismal, with a reported median PFS of 4.1 months and OS of 6.4 months [87]. Chemotherapy-based treatment is mostly ineffective, so these patients should be considered candidates for clinical trials with novel therapies (Fig. 2).

## Immune checkpoint blockade

The first clinical evidence that immune checkpoint blockade might be effective was obtained in seven patients with relapsed/refractory NK/T-cell lymphoma failing asparaginase-based regimens and allogeneic HSCT [88]. Treatment with the anti-PD1 antibody pembrolizumab resulted in an ORR of 100%, with five patients achieving CR after a median of seven cycles of treatment [88]. In another study, four of seven patients with relapsed/refractory disease responded to pembrolizumab treatment [89]. Among the two cases of CR, one patient remained in remission after eighteen cycles of treatment. However, there did not appear to be a correlation between PD-L1 expression on lymphoma cells and response to treatment [33, 88, 89]. Similar to pembrolizumab, the anti-PD1 antibody nivolumab was also reported to be effective at low doses in relapsed/refractory NK/T-cell lymphoma [90], with all three treated patients showing response, one of whom remaining in continuous CR after nine cycles of treatment. Sintilimab, another anti-PD1 antibody, was evaluated in 28 patients with relapsed/refractory NK/T-cell lymphoma [91]. The ORR was 67.9% and the 2-year OS was 78.6%. The use of avelumab, an anti-PD-L1 antibody, had been studied in a prospective phase II study.^93^ The ORR was 38% with a CR rate of 24%. Five patients had a durable response after a median of eighteen cycles of treatment. PD-L1 expression on lymphoma cells correlated with treatment response [92].

In summary, immune blockade of the PD1/PD-L1 axis represents a safe and effective treatment for relapsed/refractory NK/T-cell lymphoma. However, factors predictive of response are still largely undefined. Hence, immune checkpoint inhibition in NK/T-cell lymphoma should continue to be investigated in clinical trials. Finally, future studies of its combination with chemotherapy or other novel treatment are warranted.

## Other immunotherapies and cellular therapy

NK/T-cell lymphoma cells express CD30 and CD38, both of which had been explored as therapeutic targets. The anti-CD30 antibody conjugate brentuximab vedotin (BV) had been reported to be efficacious in two patients with relapsed/refractory NK/T-cell lymphomas [93, 94]. However, formal studies of BV in NK/T-cell lymphomas have not been conducted. Anecdotal evidence suggested that the anti-CD38 antibody daratumumab might be effective for relapsed NK/T-cell lymphoma [95]. In a formal phase 2 study in relapsed/refractory patients, however, results of daratumumab were disappointing, showing an ORR of merely 25% with no CR [96].

Another immunotherapeutic strategy is adoptive cellular therapy using autologous EBV-specific cytotoxic T-cells (CTL). In a phase 2 study of relapsed NK/T-cell lymphomas, autologous EBV-specific CTL was successfully generated in 32/47 cases, with fifteen patients subsequently administered the product [97]. The ORR was 50% (CR: 30%), with a median PFS of 12.3 months. The logistic complexity, high production failure rate (32%), and long duration to product availability (about 25 days) limit the clinical usefulness of this approach.

## Novel drugs

Chidamide is an orally active inhibitor of histone deacetylases 1, 2, 3, and 10 (HDACi) [27]. In three studies involving 115 cases of relapsed/refractory NK/T-cell lymphomas [98–100], chidamide treatment led to an ORR of 38% (CR: 16%). Alisertib, an aurora kinase A inhibitor, was used in five cases of relapsed/refractory NK/T-cell lymphomas as part of two studies [101, 102], with only one case (20%) showing a partial response. Other drugs approved in T-cell lymphomas, including pralatrexate and romidepsin [103], had been tested in too few cases of NK/T-cell lymphomas for their efficacies to be defined.

## Conclusions and perspectives

The past decade has seen significant improvement in the treatment of stage I/II NK/T-cell lymphomas, with the majority of patients expecting a cure with asparaginase-based regimens in combination with radiotherapy. However, the management of stage III/IV, and relapsed and refractory NK/T-cell lymphomas remains challenging. In addition to genomic analysis, in-depth studies on EBV-associated oncogenesis and anti-tumour immunity at single-cell resolution may offer novel multi-prong approaches of targeting the lymphoma cells and their microenvironment. Because NK/T-cell lymphomas are relative uncommon even in regions where they are more prevalent, multicentre clinical trials should also be established to guide future mechanism-based treatment in the era of precision medicine.

## Data Availability

Not applicable.

## Abbreviations

NK: Natural killer
ε: Epsilon
TCR: T-cell receptor
EBV: Epstein–Barr virus
ISH: In situ hybridization
EBER: EBV-encoded small RNA
WHO: World Health Organization
JAK/STAT: Janus kinase–signal transducers and activator of transcription
PD-L1/2: Programmed cell death protein ligand 1/2
LOH: Loss of heterozygosity
PD1: Protein programmed cell death protein 1
PET/CT: Positron emission tomography computed tomography
IPI: International Prognostic Index
ORR: Overall response rate
CR: Complete response
PFS: Progression-free survival
OS: Overall survival
AspaMetDex: Asparaginase, methotrexate, dexamethasone
DDGP: Dexamethasone, cisplatin, gemcitabine, pegaspargase
DeVIC: Dexamethasone; etoposide; ifosfamide; carboplatin
DICE-L-asp: Dexamethasone, ifosfamide, cisplatin, etoposide, L-asparaginase
GELAD: Gemcitabine, etoposide, pegaspargase, dexamethasone
GELOX: Gemcitabine, L-asparaginase, oxaliplatin
LVP: L-asparaginase, vincristine, prednisolone
MEDA: Methotrexate, etoposide, dexamethasone, pegaspargase
MESA: Methotrexate, etoposide, dexamethasone, pegaspargase
P-GEMOX: Pegaspargase, gemcitabine, oxaliplatin
SMILE: Dexamethasone, methotrexate, ifosfamide, L-asparaginase, etoposide
VIDL: Etoposide, ifosfamide, dexamethasone, L-asparaginase
VIPD: Etoposide, ifosfamide, cisplatin, dexamethasone
HSCT: Haematopoietic stem cell transplantation
CNS: Central nervous system
CTL: Cytotoxic T-cells
HDACi: Histone deacetylase inhibitor
